# Identification of Genomewide Alternative Splicing Events in Sequential, Isogenic Clinical Isolates of Candida albicans Reveals a Novel Mechanism of Drug Resistance and Tolerance to Cellular Stresses

**DOI:** 10.1128/mSphere.00608-20

**Published:** 2020-08-12

**Authors:** Suraya Muzafar, Ravi Datta Sharma, Abdul Haseeb Shah, Naseem A. Gaur, Ujjaini Dasgupta, Neeraj Chauhan, Rajendra Prasad

**Affiliations:** a Amity Institute of Integrative Sciences and Health, Amity University Gurgaon, Gurgaon, India; b Public Health Research Institute, New Jersey Medical School, Rutgers, The State University of New Jersey, Newark, New Jersey, USA; c Department of Bioresources, University of Kashmir, Srinagar, Jammu and Kashmir, India; d Yeast Biofuel Group, International Centre for Genetic Engineering and Biotechnology, New Delhi, India; University of Georgia

**Keywords:** *Candida albicans*, alternative splicing, SOD3, amphotericin B, menadione

## Abstract

Alternative splicing (AS)—a process by which a single gene gives rise to different protein isoforms in eukaryotes—has been implicated in many basic cellular processes, but little is known about its role in drug resistance and fungal pathogenesis. The most common human fungal pathogen, Candida albicans, has introns in 4 to 6% of its genes, the functions of which remain largely unknown. Here, we report AS regulating drug resistance in C. albicans. Comparative RNA-sequencing of two different sets of sequential, isogenic azole-sensitive and -resistant isolates of C. albicans revealed differential expression of splice isoforms of 14 genes. One of these was the superoxide dismutase gene *SOD3*, which contains a single intron. The *sod3*Δ/Δ mutant was susceptible to the antifungals amphotericin B (AMB) and menadione (MND). While AMB susceptibility was rescued by overexpression of both the spliced and unspliced *SOD3* isoforms, only the spliced isoform could overcome MND susceptibility, demonstrating the functional relevance of this splicing in developing drug resistance. Furthermore, unlike AMB, MND inhibits *SOD3* splicing and acts as a splicing inhibitor. Consistent with these observations, MND exposure resulted in increased levels of unspliced *SOD3* isoform that are unable to scavenge reactive oxygen species (ROS), resulting in increased drug susceptibility. Collectively, these observations suggest that AS is a novel mechanism for stress adaptation and overcoming drug susceptibility in C. albicans.

**IMPORTANCE** The emergence of resistance in Candida albicans, an opportunistic pathogen, against the commonly used antifungals is becoming a major obstacle in its treatment. The necessity to identify new drug targets demands fundamental insights into the mechanisms used by this organism to develop drug resistance. C. albicans has introns in 4 to 6% of its genes, the functions of which remain largely unknown. Using the RNA-sequencing data from isogenic pairs of azole-sensitive and -resistant isolates of C. albicans, here, we show how C. albicans uses modulations in mRNA splicing to overcome antifungal drug stress.

## Abstract (precis)

The emergence of resistance in Candida albicans, an opportunistic pathogen, against the commonly used antifungals is becoming a major obstacle in its treatment. The necessity to identify new drug targets demands fundamental insights into the mechanisms used by this organism to develop drug resistance. C. albicans has introns in 4 to 6% of its genes, the functions of which remain largely unknown. Using the RNA-sequencing data from isogenic pairs of azole-sensitive and -resistant isolates of C. albicans, here, we show how C. albicans uses modulations in mRNA splicing to overcome antifungal drug stress.

## INTRODUCTION

Candida albicans is the most common opportunistic human fungal pathogen and has a high mortality rate in patients who are severely immunocompromised (1–3). Candidiasis is most often caused by C. albicans, but there have been recent reports of other non-*albicans* species causing significant disease (4). The increasing use of antifungal agents has led to the development of resistance to the currently available antifungals (5).

*Candida* species have a repertoire of mechanisms that account for acquired antifungal drug resistance. The best-documented are increased drug efflux and compromised drug import. Other mechanisms of drug resistance in *Candia albicans* include alteration or overexpression of drug targets and chromosome duplications (6–8). Furthermore, recent reports have also implicated fungal stress response pathways to oxidative, osmotic, thermal, nitrosative, and nutrient limitation stressors as important mediators of drug resistance (9). The development of antifungal drug resistance is a complex multifactorial process that relies on both host and fungal factors. Due to the clinical importance of candidiasis, there is an urgent need to identify new drug targets and therefore new antifungal drugs for the treatment of high-risk patients.

Alternative pre-mRNA splicing (AS) is an important regulatory mechanism that expands the eukaryotic proteome, enabling cellular responses to cope with diverse environmental challenges (10–12). Most of the eukaryotic genes contain introns that are removed posttranscriptionally. The total number of introns in any given eukaryotic genome varies between different species, being higher in metazoans than in the lower eukaryotes like yeasts. Alternative splicing (AS) in metazoans has been well documented; impacts tissue-specific gene expression, gene function, and protein localization; and thereby has been implicated in many diseases (11, 13, 14). However, the fungal proteome is comparatively less subject to functional diversification by AS (15, 16). The percent proportion of introns in fungal genome(s) also varies widely, ranging from 2% to 6% in Saccharomyces cerevisiae, Candida glabrata, and C. albicans, whereas the basidiomycete human fungal pathogen Cryptococcus neoformans contains an intron in 99% of its genes, with intron retention (IR) being the most common form of AS in this species (16–20).

The biological impact of AS in fungi remains largely unexplored, although there are several instances in yeasts where functional AS has been observed (15, 21–23). For example, a serine/threonine phosphatase-encoding gene, *PTC7*, in Saccharomyces cerevisiae contains a single intron that can be alternatively spliced to produce two distinct proteins that differentially localize and function in the cell (21). Similarly, the cellular localization of a central carbon metabolism enzyme encoded by the malate dehydrogenase gene (*ylMDH2*) is governed by functional AS, which generates cytosolic or peroxisome-localized isoforms in Yarrowia lipolytica (22). The dual localization of 6-phosphogluconate dehydrogenase encoded by *GND1* in C. albicans is also directed by AS, resulting in cytosolic and peroxisome-localized isoforms (23). In addition to protein localization, AS is also regulated by different environmental stresses (16, 21, 22, 24–26). In C. albicans, the *RPL30* gene encoding a ribosomal protein of the large subunit undergoes AS, and the isoform preference is influenced by growth temperature, while AS of a sporulation-regulated gene (*SPR28*) is responsive to the α-factor mating pheromone (27). Recently, C. albicans *SOD3* and *CAN3* were also predicted to undergo AS, but its impact on cellular physiology remains largely unknown (28). C. glabrata employs stress-specific AS to increase the number of proteins in the *EPA* adhesin gene family (29). These examples demonstrate that AS in fungi contributes substantially to diversity in their proteome landscape. However, none so far have implicated a role in the development of drug resistance. Since the impact of AS on the development of drug tolerance in *Candida* spp. remains unexplored, we rationalized that an understanding of posttranscriptional regulation of development of drug resistance may open new avenues for drug responses by *Candida* spp.

## RESULTS

### Intron landscape in azole-susceptible and -resistant isogenic isolates of Candida albicans.

Various bioinformatics approaches have been employed to identify the total number of introns in the C. albicans genome (27, 30). The most comprehensive landscape of introns was generated by Bruno et al. (31), where they identified 426 introns in C. albicans (SC5314). To assess the potential functional role of AS in drug resistance in C. albicans, we used an existing resource of genetically matched azole-sensitive and -resistant sequential clinical isolates of C. albicans (32). These 17 isogenic identical isolates (TW1 to -17) were recovered from a single HIV-infected patient over a period of 2 years and showed increasing resistance (over 200-fold) over that time to the antifungal fluconazole (FLC) (32). It provided an ideal platform to monitor sequential changes associated with the development of drug resistance in an isogenic background. The comparison between sequential, matched isolates excluded the possibility of any changes due to clonal variation.

**FIG 1:**
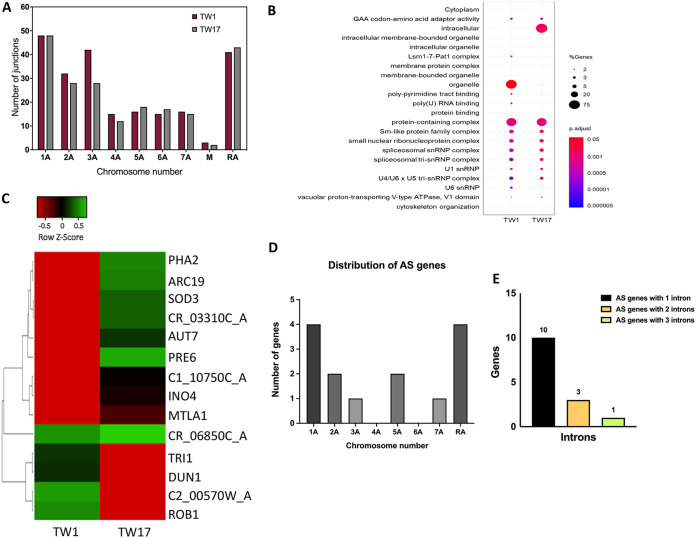
Alternative splicing events in isogenic clinical isolates of Candida albicans. (A) Chromosomal distribution of the total junctions identified in TW1 and TW17 isolates. (B) GO analysis of all the intron-containing genes identified, plotted using GG Plots. (C) Heatmap showing the differential expression of junctions between TW1 and TW17 cells. The 14 junctions correspond to 14 different genes. Count-per-million (cpm) values were used to plot this map in Heat mapper, an online software program to generate heatmaps. (D) Chromosomal distribution of AS junctions. (E) Number of introns in AS genes. The majority of the genes have only a single intron.

We performed transcriptome sequencing (RNA-Seq) analysis with early (TW1 and -2), middle (TW8 and -9), and late (TW16 and -17) isolates and identified a total of 364 junctions (exon-exon junction) in all the isolates, which is slightly lower than previous reports (27, 31). This may be due to different C. albicans strains and growth conditions used in our experiments. The intronic sequences corresponding to these junctions are given as supplemental material (see Data Set S1). The number of junctions in TW1 and TW17 is plotted along with their chromosomal location in Fig. 1A. The gene ontology (GO) analysis of total junctions between the TW1 (azole-sensitive) and TW17 (azole-resistant) strains revealed that the majority of the intron-containing genes were similar between the two isolates. However, in the TW17 isolate genes belonging to the GO term “intracellular” category were found to be more enriched than in the TW1 strain, where “organelle” category genes were preferentially enriched. The “organelle” category enriched in the TW1 strain includes genes like *ZCF24*, *PSF2*, *CLA4*, *SNP3*, *ROB1*, *GWT1*, *APN1*, *GTT11*, and C2_00570W_A. These genes are the components of the GINS, RSC (Remodels the Structure of Chromatin), and snRNP (small nuclear ribonucleoprotein) complexes. Out of these genes, some also have the molecular function of zinc ion binding. The “intracellular” category enriched in the TW17 strain includes genes like *UBP6*, *SOD3*, *AUT7*, *APS3*, *INO4*, *MTLA1*, C5_04860C_A, *LMO1*, *HHO1*, *ARC19*, *ECM4*, *PRE6*, *RHO3*, *TPS3*, and *ECM4.* The majority of these genes are predicted to be localized to the nucleus and have DNA binding activity. The gene-enriched categories in both azole-resistant and -sensitive isolates (corrected *P* value <0.05) are plotted in Fig. 1B. Details related to each category are shown in the supplemental material (Data Set S3).

10.1128/mSphere.00608-20.8 DATA SET S1 List of total junctions identified. Download Data Set S1, XLSX file, 0.3 MB. Copyright © 2020 Muzafar et al. 2020 Muzafar et al. This content is distributed under the terms of the Creative Commons Attribution 4.0 International license.

### Differential expression of select junctions in resistant isolates.

**FIG 2:**
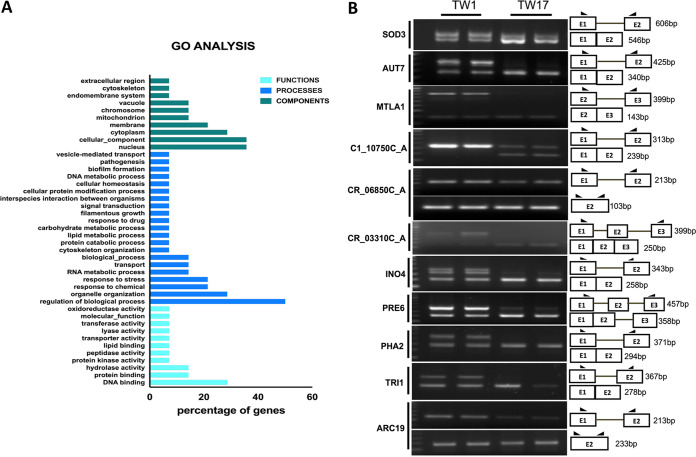
Validation of AS events. (A) Gene ontology (GO) analysis was performed using GO Term Finder on the Candida Genome Database with the AS gene list. (B) Validation of AS events using RT-PCR. The two lanes for each sample represent the biological replicates.

The gradual change in the intron landscape obtained between resistant and sensitive isogenic pairs of strains of C. albicans led us to investigate the RNA-Seq data in greater depth. For pairwise comparative junction analysis (see Materials and Methods), we used only TW1 as the most susceptible and TW17 as the most resistant isogenic pair. Our analysis revealed differential expression of junctions and predicted 14 AS events specific to the resistant TW17 isolate compared to the sensitive TW1 isolate. All the 14 AS events are intron retention (IR) events involving partial or complete retention of introns with most of the genes containing only one intron (Fig. 1C to E). Figure 1C shows the differential expression of exon-exon junctions between the sensitive and resistant isolates. Interestingly, a relatively higher number of the AS genes were found to be located on chromosomes 1A and RA (Fig. 1D). Most of the AS genes in C. albicans contain a single intron (Fig. 1E). A gene ontology (GO) analysis using the Candida Genome Database (CGD) for biological processes and functions affected by the predicted AS events showed enrichment for DNA and protein binding, hydrolase activity, lipid binding, transporter activity, and the oxidoreductase group of genes, among others (Fig. 2A). To establish the validity of the predicted AS events, we validated all predicted intron retention (IR) events in genes critical for response to chemicals and stress (*ARC19*, *SOD3*, and *AUT7*), lipid and carbohydrate metabolic processes (*INO4*), transport processes (*AUT7* and C110750C_A), protein catabolic process (*PRE6*), cytoskeletal organization (*ARC19*), pathogenesis-related function (*MTLA1*), and open reading frames (ORFs) with unknown functions (C1_10750C_A, CR_06850C_A, CR_03310C_A, and *TRI1*) (Table 1). All AS genes showed a similar pattern in their differential expression between unspliced and spliced isoforms (Fig. 2B). Specifically, in all validated genes, the IR isoform was more highly expressed in the sensitive strain TW1, whereas the spliced isoform was more highly expressed in the resistant strain TW17. As with most IR transcripts, the IR events led to inclusion of premature termination codons (PTC), likely leading to reduced levels of the protein-coding transcript.

**TABLE 1:** List of AS genes with the position of the alternatively spliced intron along with the *P* values in Candida albicans (TW1 and TW17) strains

Serial no.	Gene	*P* value	Intron position
1	*ARC19*	4.38397E−06	i1 (85–4)
2	*PHA2*	3.58946E−05	i1 (130–52)
3	CR_03310C_A	6.26674E−05	i1 (706–648)
4	*SOD3*	6.87716E−05	i1 (105–165)
5	*ROB1*	0.000221735	i1 (47–107)
6	*DUN1*	0.000326354	i1 (118–22)
7	*AUT7*	0.000339926	i1 (34–118)
8	CR_06850C_A	0.000663398	i1 (73–4)
9	C2_00570W_A	0.000983209	i1 (34–107)
10	*TRI1*	0.00115384	i3 (736–822)
11	*INO4*	1.22467E−05	i1 (217–133)
12	*PRE6*	8.54106E−05	i1 (35–130)
13	C1_10750C_A	0.000239973	i1 (184–258)
14	*MTLA1*	0.000427415	i2 (639–894)

### Isogenic drug-resistant (Gu5) and -sensitive (Gu4) pairs also display AS.

To determine whether differential AS is a common mechanism used by C. albicans to acquire drug resistance, we also explored AS events in another genetically matched pair of clinical isolates. For this, we used isogenic azole-susceptible Gu4 (MIC of 3.12 μg/ml) and azole-resistant Gu5 (MIC of >100 μg/ml) isolates of C. albicans recovered from an AIDS patient with oropharyngeal candidiasis (OPC) (33). Analysis of RNA-Seq data of Gu4 and Gu5 (Data Set S2) for junctions that are differentially expressed revealed eight genes (*NOP1*, *PGA18*, *LTP1*, *C1_10750C_A*, *MUQ1*, *CTA24*, *CTA2*, and *SOD3*) undergoing AS events (Table 2). Except for *SOD3* and *C1_10750C_A*, all other genes were different from those recorded between TW1 and TW17. Thus, a different isogenic azole-susceptible Gu4 and azole-resistant Gu5 pair also displayed differential AS events, but in a unique set of genes. Given that *SOD3* was differentially spliced in both sets of clinical isolates, and its known role as a reactive oxygen species (ROS) scavenger in *Candida* cells, we chose to focus on *SOD3* to evaluate the potential biological effect of the AS-based preference of isoform expression between sensitive and resistant isolates.

**TABLE 2:** List of predicted AS genes with intron position along with *P* values in Candida albicans (Gu4 and Gu5) strains

Serial no.	Gene	*P* value	Intron position
1	*NOP1*	6.93788E-06	154–199^*a*^
2	*PGA18*	0.000233241	1181–1130
3	*LTP1*	0.00037349	I-1 (228–62)
4	C1_10750C_A	0.000377641	I-1 (256–182)
5	*MUQ1*	0.000377641	134–74
6	*CTA24*	0.000585666	315–426
7	*CTA2*	0.00074396	315–396^*a*^
8	*SOD3*	0.000767368	105–165

a No intron annotation in CGD so far (novel introns).

10.1128/mSphere.00608-20.9 DATA SET S2 List of junctions (undergoing AS events) that are differentially expressed in Gu4 and Gu5 strains. Download Data Set S2, XLSX file, 0.01 MB. Copyright © 2020 Muzafar et al. 2020 Muzafar et al. This content is distributed under the terms of the Creative Commons Attribution 4.0 International license.

10.1128/mSphere.00608-20.10 DATA SET S3 The gene ontology (GO) analysis of total junctions between the TW1 (azole-sensitive) and TW17 (azole-resistant) strains. Download Data Set S3, XLSX file, 0.01 MB. Copyright © 2020 Muzafar et al. 2020 Muzafar et al. This content is distributed under the terms of the Creative Commons Attribution 4.0 International license.

### Alternative splicing responds to *in vitro* stresses.

**FIG 3:**
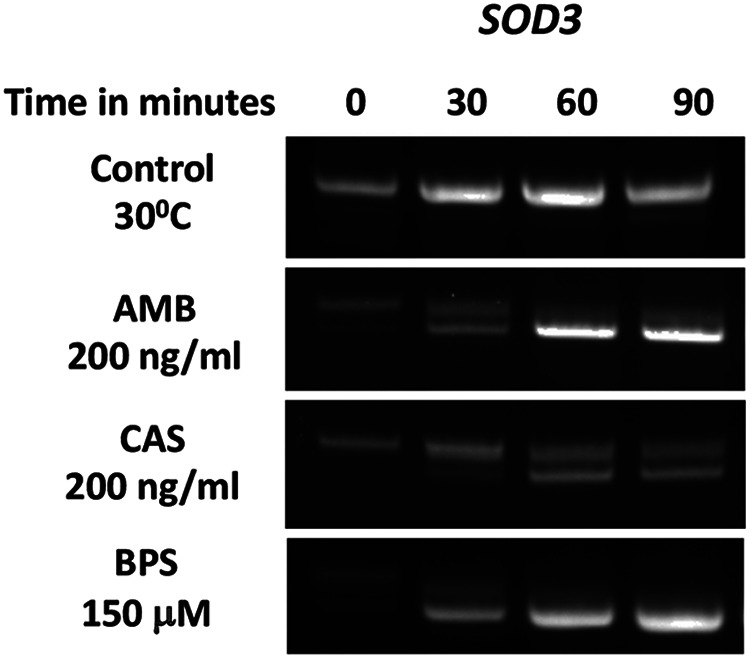
Changes in *SOD3* splicing in response to *in vitro* stresses. Gel image showing differential isoform expression in *SOD3* isoform under different stress conditions. Log-phase cells were exposed to different stresses. Drug stresses (AMB and CAS), and metal deficiency (BPS), were applied as described in Materials and Methods. Samples were collected at regular time intervals beginning with time zero and then collected after every 30-minute interval (0, 30, 60, and 90 min). Each experiment was repeated three times.

We considered that differential AS in a subset of genes was a strategy employed by the resistant strains to overcome drug sensitivity. Differential isoform expression of AS genes in the resistant strain may help them to combat the stress by generating a higher proportion of spliced (i.e., functional) protein product, as in the case of *SOD3* (Fig. 2B). This prompted us to analyze whether such differentially expressed, splice isoforms are also generated in other *in vitro* stress situations. For this, we exposed TW1 cells to one of two antifungal compounds, amphotericin B (AMB) and caspofungin (CAS), or grew them under iron-depleting conditions induced by a chelator, bathophenanthroline disulfonate (BPS), in the growth medium. We observed a significant increase in the spliced isoform of *SOD3* beyond 60 min induced by AMB and by BPS and the total absence of the unspliced form by 90 min (Fig. 3). This provides strong evidence of an acute need for Sod3 protein to overcome these drug-induced stress conditions.

### *SOD3* levels impact drug susceptibility in Candida albicans.

The C. albicans genome contains six different superoxide dismutase (SOD) genes (*SOD1* to *-6*). Among these, *SOD1* and *SOD3* are cytosolic, *SOD2* is mitochondrial, and *SOD4*, *5*, and *6* are cell surface (glycosylphosphatidylinositol [GPI])-anchored superoxide dismutases (34, 35). The primary function of the SODs in *Candida* spp is to detoxify both ROS generated from its cellular metabolism and the ROS it encounters from the host immune defenses (34, 36). *SOD4*, *5*, and *6* are especially implicated in detoxifying ROS generated by macrophages, which helps *Candida* evade host immune surveillance (35, 37–39). C. albicans is better equipped to deal with oxidative stresses as it has two cytosolic SODs (*SOD1* and *SOD3*), whose expression is reciprocally regulated (40, 41). A report showed upregulation of *SOD* genes in miconazole-treated sessile cells of C. albicans biofilms (34). Additionally, *SOD1* and *SOD5* genes are also shown to be important for the virulence of C. albicans (39, 42).

**FIG 4:**
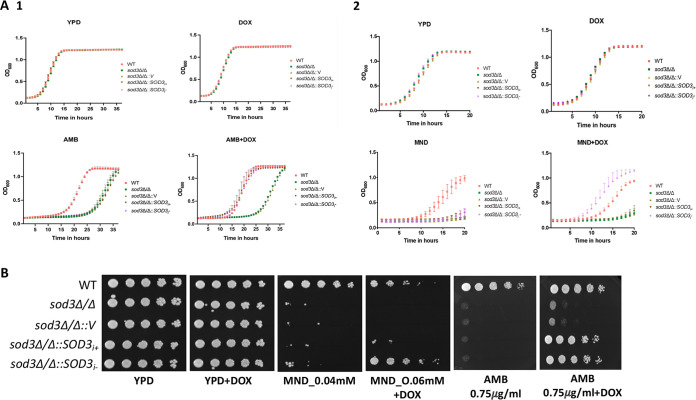
Growth properties of *sod3*Δ/Δ and overexpressing (*sod3*Δ/Δ::*SOD3_i_*_+_) or (*sod3*Δ/Δ::*SOD3_i−_*) isoforms. (A) (Panel 1) Growth curves of *sod3*Δ/Δ knockout and individual isoform (*sod3*Δ/Δ::*SOD3_i_*_+_ or *sod3*Δ/Δ::*SOD3_i−_*) overexpressing strains in YPD and AMB (0.2 μg/ml) in the presence and absence of doxycycline (DOX; 50 μg/ml). The DOX-only condition was also used as a control. (Panel 2) Strains in YPD and MND (0.1 mM) in the presence and absence of doxycycline (DOX; 50 μg/ml). (B) The susceptibility of *sod3*Δ/Δ and the individual isoform (*sod3*Δ/Δ::*SOD3_i_*_+_ *or sod3*Δ/Δ::*SOD3_i−_*) overexpressing strains to AMB and MND was tested by spot assays on solid agar medium under similar conditions as in panel A, confirming the growth curve results.

Since our study was mainly focused on the differential expression of isoforms of genes arising due to intron retention between azole-sensitive and -resistant clinical isolates of C. albicans, we analyzed intron sequences of all SOD genes. Only *SOD1* and *SOD3* possess intronic sequences, wherein *SOD1* has a comparatively longer intron of 244 bp compared to the 60-bp intron of *SOD3* (40, 42, 43). Our observation of *SOD3* specifically manifesting AS led us to explore whether *SOD3* has any role in influencing the drug susceptibility of *Candida* cells. For this, extensive drug susceptibility assays in the presence of different drugs were performed in a *sod3*Δ/Δ homozygous mutant and its wild-type (WT) parent strains. The susceptibility of all tested drugs, which included azoles such as fluconazole, voriconazole, itraconazole, miconazole, and echinocandins, did not change in *sod3*Δ/Δ mutant cells compared to the WT strain. However, *sod3*Δ/Δ mutant cells exhibited increased susceptibility to the polyene drug AMB and to menadione (MND) (Fig. 4). Notably, the susceptibility of *sod3*Δ/Δ mutant cells to another quinone (1,2-naphthoquinone) did not change (Fig. S1). Given that only *SOD1* and *SOD3* have introns, we also checked the susceptibility of *sod1*Δ/Δ mutants toward different drugs. The *sod1*Δ/Δ mutant was also susceptible to AMB and MND and did not show any change in sensitivity to azole or 1,2-naphthoquinone (Fig. S2). Collectively, these data imply that in addition to its predicted role in protection against oxidative stress under physiological conditions, the C. albicans *SOD3* gene also plays an important role in resistance to antifungal drugs.

10.1128/mSphere.00608-20.1 FIG S1 Susceptibility of *sod3Δ/Δ* and other strains to MND and NQN (1,2-naphthoquinone). (A) C. albicans strains SC5314, Gu4, Gu5, TW1, and TW17 were spotted on solid YPD plates containing indicated concentrations of MND and NQN. Both the azole-sensitive strains (Gu4 and TW1) show more susceptibility to MND than the resistant strains (Gu5 and TW17), whereas Gu4 and TW1 are slightly more sensitive to NQN than Gu5 and TW17. (B) Spotting of WT, *sod3*Δ/Δ, and overexpression strains on MND and NQN. The *sod3*Δ/Δ strain does not show any change in susceptibility to NQN. Download FIG S1, TIF file, 0.3 MB. Copyright © 2020 Muzafar et al. 2020 Muzafar et al. This content is distributed under the terms of the Creative Commons Attribution 4.0 International license.

10.1128/mSphere.00608-20.2 FIG S2 Susceptibility of *sod1Δ/Δ* and *sod3Δ/Δ* strains to different drugs. The *sod1Δ/Δ* and *sod3Δ/Δ* strains are susceptible to MND and AMB while showing no change to azoles (fluconazole [FLU] and voriconazole [VRZ]), caspofungin (CAS), and 1,2-naphthoquinone (NQN). Download FIG S2, TIF file, 0.4 MB. Copyright © 2020 Muzafar et al. 2020 Muzafar et al. This content is distributed under the terms of the Creative Commons Attribution 4.0 International license.

### Only the spliced isoform of *SOD3* complements MND susceptibility.

To determine the role of AS in the *SOD3* gene, we engineered C. albicans to overexpress either the spliced or unspliced isoforms of *SOD3* (as described in Materials and Methods). Since both the isoforms were cloned under a TET-regulatable promoter, the experiments were done in the presence of doxycycline as indicated. Overexpression of the green fluorescent protein (GFP)-tagged Sod3 fusion protein with individual isoforms (*sod3Δ/Δ::SOD3*_I+_ [unspliced] or *sod3*Δ/Δ::*SOD3*_I−_ [spliced]) was confirmed by Western blotting (Fig. S3). These strains along with the *sod3Δ/Δ* mutant, and WT cells, were then subjected to drug susceptibility tests in the presence and absence of doxycycline. The overexpression of spliced isoform (*sod3*Δ/Δ::*SOD3*_I−_) rescued the susceptibility to both AMB and MND. However, the intron-containing (unspliced) isoform (*sod3*Δ/Δ::*SOD3*_I+_) complemented only AMB susceptibility, while MND susceptibility remained largely unchanged. The *sod3*Δ/Δ::*V* strain is the homozygous mutant with the empty vector added back. This strain was created by transforming the *sod3*Δ/Δ null mutant with the empty plasmid vector. This strain was included as a control in all experiments. We confirmed these results by both microdilution liquid and spot assays. We did not observe any change in susceptibility to doxycycline alone of all C. albicans strains used in the assay (Fig. 4A and B).

10.1128/mSphere.00608-20.3 FIG S3 Western blot image of Sod3 overexpression strains showing expression of GFP-tagged Sod3 protein in the presence of doxycycline (uncropped). The *sod3*Δ/Δ::V strain (homozygous mutant with empty vector added back) was used to check GFP expression. Strains were grown in the presence or absence of doxycycline. GFP-fused Sod3 protein was expressed only in the presence of doxycycline. Download FIG S3, TIF file, 0.2 MB. Copyright © 2020 Muzafar et al. 2020 Muzafar et al. This content is distributed under the terms of the Creative Commons Attribution 4.0 International license.

### The spliced isoform of *SOD3* decreases ROS when exposed to MND.

**FIG 5:**
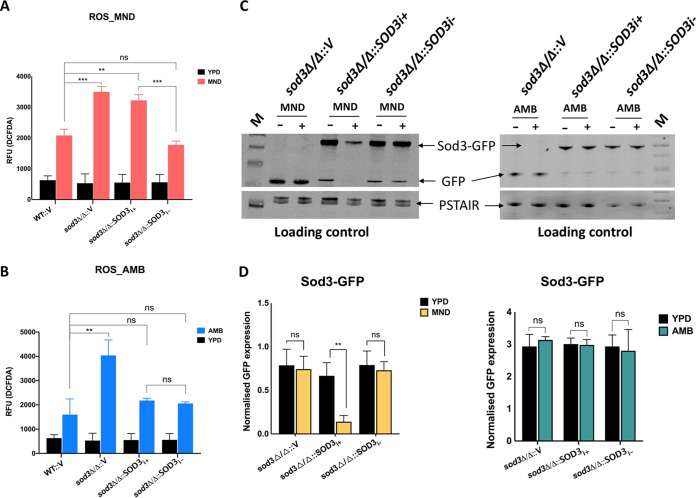
ROS production in *sod3*Δ/Δ and unspliced (*sod3*Δ/Δ*SOD3_i_*_+_) and spliced (*sod3*Δ/Δ::*SOD3_i−_*) isoform overexpressing strains. (A and B) Defective ROS scavenging of unspliced isoform (*sod3*Δ/Δ::*SOD3_i_*_+_) upon MND treatment. Cells were treated with MND (0.1 mM) or AMB (0.2 μg/ml) and then washed with PBS and incubated with H_2_DCFDA, a fluorescent dye. Fluorescence intensities were measured with excitation at 495 nm and emission at 520 nm in a 96-well plate using a microplate reader. The values represent means from three biological replicates. The *sod3*Δ/Δ::V strain is the homozygous mutant with the empty plasmid vector added back. This strain was included as a control in all experiments. Error bars represent standard deviation. Statistical analysis was performed using unpaired two-tailed *t* test (**, *P* < 0.01; ***, *P* < 0.001; ns, nonsignificant). (C and D) Western blots showing expression of Sod3-GFP fusion protein in the presence of MND and AMB. (C) Sod3-GFP expression decreases significantly in the strain expressing unspliced isoform (*sod3*Δ/Δ::*SOD3_i_*_+_) in the presence of MND while no significant difference is detected in the expression of Sod3-GFP in the presence of AMB. Lane M, size markers. (D) Protein bands were quantified using ImageJ, normalized to loading control (anti-PSTAIR), and plotted using GraphPad Prism. The values represent the mean from three biological replicates. Error bars represent standard deviation. Statistical analysis was performed using unpaired two-tailed *t* test (**, *P* < 0.01; ***, *P* < 0.001; ns, nonsignificant).

We next evaluated why the strain expressing the unspliced *SOD3* isoform was unable to complement MND susceptibility. Since *SOD3* is a ROS scavenger and both AMB and MND are known to induce ROS production (44–48), we measured ROS levels in these strains in the presence and absence of AMB and MND. We found that both isoforms of the *SOD3* gene (*sod3*Δ/Δ::*SOD3*_I+_ or *sod3*Δ/Δ::*SOD3*_I−_) were able to reduce ROS levels when cells were exposed to AMB. In contrast, only the spliced isoform was able to reduce ROS levels in the presence of MND (Fig. 5A and B). We next evaluated Sod3 protein expression levels in the presence and absence of AMB and MND by Western blotting using an anti-GFP monoclonal antibody. The protein levels in the strain with the unspliced isoform of *SOD3* were significantly reduced compared to the one with the spliced isoform in the presence of MND, while they remained unchanged in AMB-exposed cells. (Fig. 5C and D). These results suggest that in the presence of MND, cells express mostly the unspliced *SOD3* isoform, which is unable to combat oxidative stress.

### Menadione inhibits pre-mRNA splicing.

**FIG 6:**
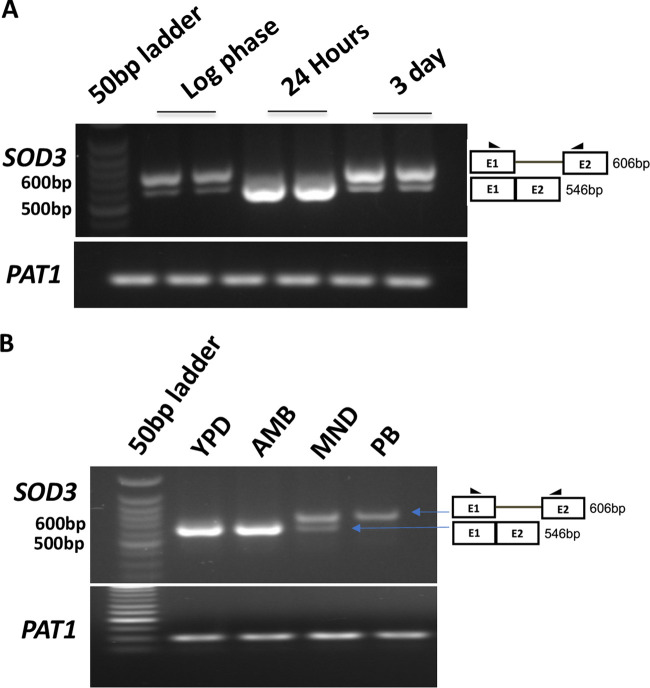
Gel images showing differential expression of *SOD3* isoforms at different growth phases in Candida albicans. (A) The unspliced isoform of *SOD3* is more prominently expressed than the spliced isoform in log-phase cells and 3-day-old stationary-phase cells, while the spliced isoform is more prominent at 24 h of growth. The two lanes for each sample represent the biological replicates. (B) Gel image showing the inhibition of *SOD3* pre-mRNA splicing by MND and splicing inhibitor pladienolide (PB) with no inhibition by AMB. PB (80 μg/ml) was included as a control in this experiment. The 24-h time point was used to check the effect of MND and AMB on *SOD3* pre-mRNA splicing.

We rationalized that when cells are exposed to AMB, the unspliced *SOD3* isoform gets spliced, but that is somehow blocked when cells are exposed to MND. Thus, we tested whether MND inhibits *SOD3* splicing by analyzing the effect of MND and AMB on pre mRNA splicing of the *SOD3* gene. Notably, earlier reports showed that the expression of *SOD3* changes during growth phases with its expression increasing in the stationary phase due to the changes in copper concentrations in the medium (40, 41). Thus, for monitoring changes in the splicing pattern of the *SOD3* gene with different growth phases, we employed the WT (SC5314) strain grown until different growth phases over a period of time and collected log-phase, 24-h, and 3-day stationary-phase cells as described in Materials and Methods. We analyzed the *SOD3* splicing pattern in cells harvested at these three growth phases and found that *SOD3* is present mostly as an unspliced isoform in log-phase cells. In contrast, the spliced isoform dominated in 24-h cultures, but levels decreased after 3 days of growth, reflecting an increase in the unspliced isoform, similar to log-phase cells. (Fig. 6A). Thus, we selected the 24-h time point, with the highest levels of spliced *SOD3* isoform, to check the effect of MND and AMB on the extent of *SOD3* splicing. The C. albicans WT cells were grown in the presence of MND and AMB for 24 h. The splicing pattern of the *SOD3* gene in the presence of AMB remained similar to that of the WT (SC5314) strain grown without any drug. However, in the presence of MND, *SOD3* splicing was inhibited, which was evident from the significantly higher ratio of the *SOD3* unspliced isoform (Fig. 6B). Pladienolide B (PB) is a known splicing inhibitor that works by blocking the SF3b splicing factor. PB (80 μg/ml) was used as a control in this experiment to test the splicing inhibition level of MND (Fig. 6B).

**FIG 7:**
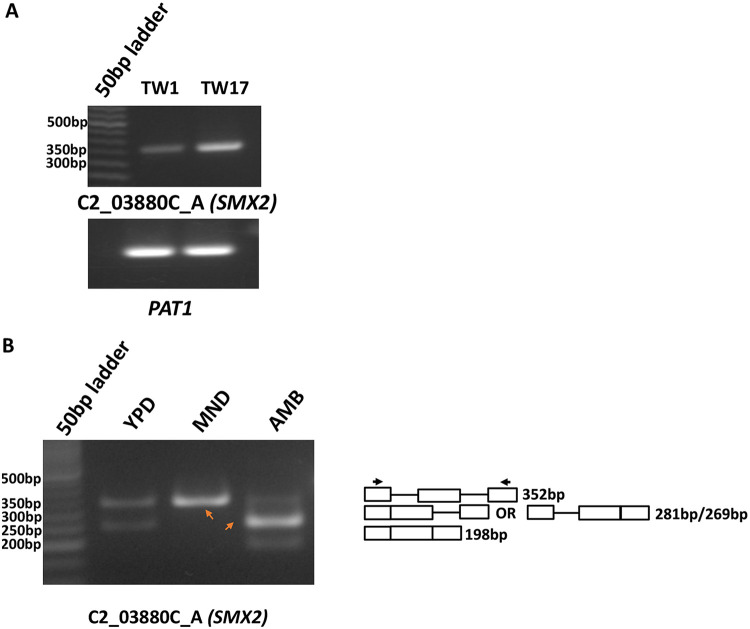
Gel images showing differential gene expression and differential isoform expression of C2_03880C_A (*SMX2*) along with quantification. (A) Differential expression of C2_03880C_A (*SMX2*) in TW1 and TW17. The experiment was performed in triplicate. Band intensity was normalized to loading control (PAT1) and plotted using GraphPad Prism. (B) Gel image showing differential isoform expression of C2_03880C_A (*SMX2*) with MND and AMB exposure. MND inhibits splicing, and only the unspliced isoform is expressed. In contrast, AMB increases *SMX2* splicing, leading to a predominance of the spliced isoform with one intron removed. The other isoform in which both the introns are removed is also visible upon AMB treatment. The orange arrows indicate different isoforms of C2_03880c_A (SMX2).

Our data suggest that the antifungal activity of MND can also be the result of its role as a splicing inhibitor. Thus, we assessed the splicing inhibition of other genes undergoing AS events in the presence of MND. We found that MND could also inhibit the splicing of some other genes undergoing AS (*PHA2*, *TRI1*, and *AUT7*) in the TW1-TW17 isogenic pair (Fig. S4A). We also evaluated the effect of MND on the splicing of C2_03880C_A, which was found to be upregulated in TW17 (Fig. 7A). C2_03880C_A is an ortholog of Saccharomyces cerevisiae *SMX2*, which is a homolog of the human SmG protein that functions as a splicing factor (49). In C. albicans, the C2_03880C_A (*SMX2*) gene is 453 bp with three introns. Interestingly, in MND-treated samples, only the unspliced isoform of the *SMX2* gene was detected, while AMB treatment induced expression of an alternatively spliced isoform with one of the introns being removed (Fig. 7B). This provides further evidence of an inhibitory role for MND on splicing.

10.1128/mSphere.00608-20.4 FIG S4 (A) Gel image showing splicing inhibition of some other genes by MND. To check whether MND splicing inhibition is limited to *SOD3* or whether it inhibits splicing of other genes as well, we selected *PHA2, TRI1*, and *AUT7* as these genes were also present in their spliced form at 24 h of growth. These genes were amplified from MND-treated samples and separated on 2% agarose gels. The expression of unspliced isoform is increased in MND-treated samples. (B) Gel images showing differential alternative splicing in Candida albicans at different growth phases. WT cells were grown, and samples were collected at different growth phases (log phase, 24 h, 48 h, and 3 days). *TRI1*, *PHA2*, *SOD3*, and *C2_03880C_A* were amplified and run on 2% agarose gels. The *TRI1* gene has two introns, and both of them are retained after 48 h of growth. The *PHA2* intron is also retained after 48 h. *SOD3* and *C2_03880C_A* are present in unspliced isoform at 3 days of growth. We could see splicing modulation with different growth phases, and introns tend to be retained in late stationary phase. *GAPDH* was used as a loading control. Download FIG S4, TIF file, 0.3 MB. Copyright © 2020 Muzafar et al. 2020 Muzafar et al. This content is distributed under the terms of the Creative Commons Attribution 4.0 International license.

## DISCUSSION

Alternative splicing (AS) along with other posttranscriptional modifications has been associated with virulence in several pathogenic organisms (50) as they allow pathogens to fine-tune gene expression in response to diverse host environments. Of the 4 to 6% introns in the C. albicans genome, some intron-bearing genes are morphology and virulence related, implying a role for AS in these processes. Most of the intron-containing genes have a single intron, and hence, intron retention (IR) is the most common type of AS in C. albicans. Our comparative RNA-Seq analysis of drug-sensitive and -resistant clinical isolates identified 364 intron junctions. However, only a subset of genes underwent differential AS between the isolates, all under the IR category. Importantly, expression of the spliced isoform of all the genes increased in the resistant strain, suggesting the functional importance of the spliced isoforms in acquired resistance. Most of the introns retained in the sensitive strains introduced a stop codon which likely prevented the expression of the fully functional protein, an analogous situation to that earlier reported in Cryptococcus neoformans, where IR presents an alternative mechanism of gene regulation to coding for different proteins (16).

We analyzed an AS event in *SOD3*, a well-known ROS scavenger that showed AS in two independent pairs of isogenic resistant and sensitive isolates. We show that differential expression of *SOD3* isoforms was not a response specific to the development of drug resistance of clinical isolates since changes in isoform preferences also occur upon *in vitro* exposure to drug and metal deficiency. This points toward modulation of AS in *SOD3* with varied outcome in response to a variety of cellular stresses. Recently, a study also showed that *SOD3* undergoes AS in response to oxidative stress in C. albicans (28).

The observed susceptibility of *sod3*Δ/Δ cells toward the polyene AMB and a quinone, MND, and the finding that it could be rescued only by the functional spliced isoform of *SOD3* highlighted the biological significance of AS in drug resistance. Increase in *SOD3* splicing implies abundance of the functional protein which is needed by cells to survive oxidative and metabolism-induced stress. Recent reports have shown that the ectopic expression of *SOD1* and *SOD3* rescues the genome instability in tetraploid C. albicans cells and could also repress glucose uptake (51, 52).

MND possesses antifungal activity, which gets augmented in the presence of a glucocorticoid betamethasone in C. albicans cells (53). Along with our report that *sod3*Δ/Δ C. albicans cells are susceptible to MND, *sod1*Δ/Δ cells are also reported to be susceptible to MND (37, 38, 42). Interestingly, the unspliced isoform of the *SOD3* gene did not fully complement *sod3*Δ/Δ susceptibility toward MND, which revealed its inhibitory effect on splicing compared to AMB, which allows for efficient splicing of the *SOD3* IR isoform to generate functional Sod3 protein. The role of MND as a splicing inhibitor has been suggested in human HEK293T cells (54); however, a similar role in yeast is not known. Our results suggest that *Candida* cells use AS as a gene regulatory mechanism to adjust to the changing environment by modulating the splicing pattern of different genes. On the other hand, antifungal drugs also use modulation of gene splicing as one of the mechanisms for their antifungal activity. Importantly, the AS event associated with *SOD3* did not modulate susceptibility to tested azoles; however, the fact that several genes including many uncharacterized ORFs undergo AS in drug-resistant isolates of C. albicans strongly suggests their possible roles remain to be assessed. Notably, the expression of C. albicans *SOD3* in the Saccharomyces cerevisiae *sod1*Δ/Δ mutant could rescue its sensitivity to redox cycling agents including MND (40).

**FIG 8:**
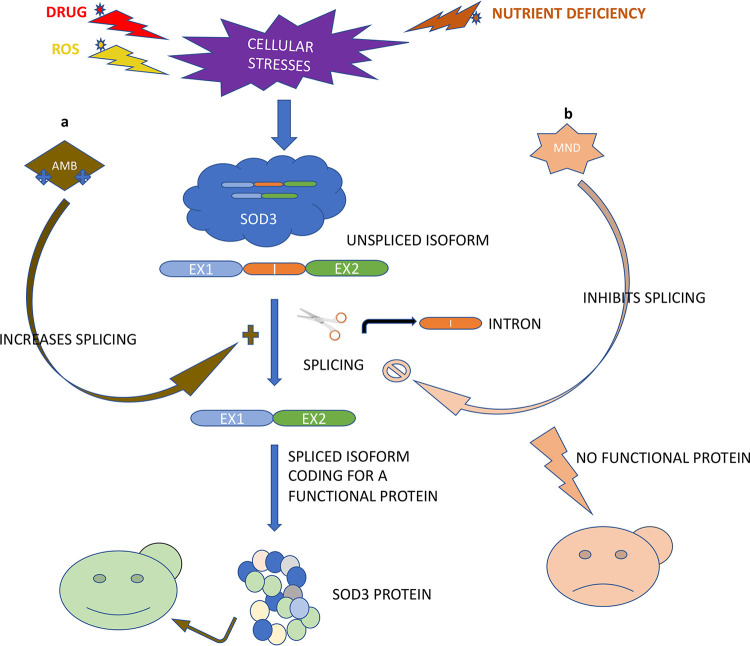
Schematic describing the effect of different cellular stresses on the splicing of the *SOD3* gene in Candida albicans. C. albicans expresses the *SOD3* gene in two isoforms, spliced isoform and an unspliced isoform (where intron is retained). When C. albicans encounters stresses like nutrient deficiency or exposure to certain drugs, which may induce ROS production, the splicing of this gene increases, producing more of the functional protein to combat stress. The differential splicing response does not follow a similar pattern. This schematic shows the opposite effects of AMB and MND on *SOD3* splicing. (a) AMB increases the *SOD3* splicing, expressing more of the spliced isoform, and thus producing more protein to combat ROS generated by this antifungal drug. (b) MND inhibits *SOD3* splicing, expressing more of the unspliced isoform which does not code for a functional protein. Lack of functional Sod3 protein furthers increases the susceptibility to this drug.

The differential expression of *SOD3* isoforms in influencing susceptibility of AMB and MND and specific splicing inhibition by MND suggests that the susceptibility of antifungal MND is probably impacted by AS of the ROS scavenger *SOD3* (Fig. 6). The role of AS in regulating the gene function in C. albicans is summarized in Fig. 8.

Introns play an important role in response to nutrient deprivation in Saccharomyces cerevisiae cells (55, 56). Our observation that splicing of *SOD3* as well as some other genes in C. albicans is inhibited as the cell enters the stationary phase (see Fig. S4B in the supplemental material) supports the role of IR under nutrient-deficient conditions (56). Reduced splicing as the cells enter into the stationary phase gives them an opportunity to enter an energy-saving mode, suggesting a new functional role for introns (57). In addition, it is possible that IR transcripts do not always get diverted to the nonsense-mediated decay pathway but instead provide a possible ready reserve or storehouse that can be efficiently spliced and translated, as needed, to combat any sudden stress encountered by the cells. Such situations occur in higher eukaryotes but of course require further elaboration in *Candida* (58). Additionally, how splicing factors are involved in regulating these modulations and how they control the splicing of different genes which may be involved in the virulence and pathogenesis or cellular stresses demand a closer look as reports show the involvement of SR (serine-arginine family of RNA binding proteins)-like proteins in virulence (59). Our study reveals the intricate interplay between splicing regulation in *Candida* and the impact of drug behavior on susceptibility of the pathogen that decides the cell fate and provides us a target that can be exploited to improve therapeutic response.

## MATERIALS AND METHODS

### Strains and growth.

Candida albicans strains were grown and maintained in yeast extract-peptone-dextrose (YPD) enriched medium. All the stocks were maintained in 25% glycerol and stored at −80°C. All the experiments were done at 30°C, unless indicated otherwise. All strains used in this study are listed in Table S1 in the supplemental material (60, 61).

10.1128/mSphere.00608-20.5 TABLE S1 List of C. albicans strains/plasmids used in this study. Download Table S1, DOCX file, 0.02 MB. Copyright © 2020 Muzafar et al. 2020 Muzafar et al. This content is distributed under the terms of the Creative Commons Attribution 4.0 International license.

### RNA extraction.

The C. albicans strains were grown in YPD medium overnight at 30°C. Cells corresponding to an optical density at 600 nm (OD_600_) of 0.1 were inoculated into fresh YPD medium. Log-phase cells (OD_600_ 0.6) were collected and washed twice with diethylpyrocarbonate (DEPC)-treated MilliQ water. Acid-washed glass beads (0.5 mm from Sigma, MO, USA) were used to break the cells. The Qiagen RNeasy minikit was used to isolate RNA from the cell pellet per the manufacturer’s instructions. The concentration of the RNA was determined using NanoDrop 2000 (Thermo Scientific, USA). The quality of the 28S and 18S rRNA on an agarose gel was used as a measure of integrity.

### cDNA synthesis.

Two micrograms of DNase I (Fermentas)-treated RNA was used for first-strand cDNA synthesis. cDNA was synthesized employing Superscript III reverse transcriptase (Invitrogen). Semiquantitative RT-PCR validation of AS events was carried out using *Taq* polymerase (New England Biolabs) and 28 to 34 cycles of PCR. The PCR products were resolved on a 2% agarose gel along with a 50-bp ladder (New England Biolabs). All the primers used in this study are described in Tables S2 and S3.

10.1128/mSphere.00608-20.6 TABLE S2 List of primers used for RT-PCR validation of the AS events in this study. Download Table S2, DOCX file, 0.02 MB. Copyright © 2020 Muzafar et al. 2020 Muzafar et al. This content is distributed under the terms of the Creative Commons Attribution 4.0 International license.

10.1128/mSphere.00608-20.7 TABLE S3 List of primers used for the construction of overexpression strains. Download Table S3, DOCX file, 0.01 MB. Copyright © 2020 Muzafar et al. 2020 Muzafar et al. This content is distributed under the terms of the Creative Commons Attribution 4.0 International license.

### RNA sequence analysis.

In order to evaluate the gradual changes in functional splicing, we selected TW1 and -2 (FLC MIC ≤1 μg/ml) as original susceptible isolates, TW8 and -9 (FLC MIC ≤8 μg/ml) as intermediate resistance, and TW16 and -17 (FLC MIC >64 μg/ml) as highly resistant to fluconazole from the collection. We subjected these pairs to RNA-Seq. To find the introns, we employed methodology similar to RNA-Seq data as was used by Bruno et al. (31, 62). For AS analysis, the raw read files of isolates were mapped to the C. albicans genome (SC5314v22) using TopHat2 software. TopHat2 software aligns raw reads in two phases. The first phase consists of aligning all reads to the reference genome. In the second phase, the remaining reads that failed to align in the first attempt are divided into two equal portions, and each portion is then aligned to the reference genome. Reads successfully aligned in the second attempt are reported as junction reads where one part is aligned to the genome and another part is aligned either upstream or downstream to the first read. Notably, the annotation of the *Candida* genome is changing continuously in the Candida Genome Database (CGD) with respect to an increase in unannotated features, and the CGD does not provide any clear annotation of introns (http://www.candidagenome.org/ [1]). In order to find the condition-specific, differentially expressed junction, we applied a linear model using the Limma platform (63) using in-house scripts in R programming language.

### Gene ontology analysis.

Gene ontology (GO) term analysis was performed through GO Slim Mapper and GO term finder using the Candida Genome Database (http://www.candidagenome.org/).

### Construction of mutant strains.

C. albicans strains expressing Sod3-GFP fusion protein were created by cloning *SOD3* spliced and unspliced isoforms individually in plasmid pNIM1 using the In-Fusion cloning kit (TaKaRa). Briefly, the *SOD3* gene was amplified with primers *SOD3A* (listed in Table S2) from cDNA. The primers were designed with overhangs from GFP and the vector backbone. The SalI restriction site was introduced into both forward and reverse primers. Both the isoforms were eluted separately from the gel and cloned into the SalI-digested pNIM1 vector. Positive clones were selected and confirmed by SalI restriction digestion. SacII- and KpnI-digested plasmids were then transformed into C. albicans *sod3*Δ/Δ as described earlier (64). Transformants were selected on plates containing 200 μ*g*/ml nourseothricin and confirmed by PCR and also by Western blotting. The *sod3*Δ/Δ mutant was a kind gift from Karl Kuchler, Medical University of Vienna, Austria.

### Growth curves.

C. albicans strains (WT and overexpression strains) were grown in YPD medium overnight at 30°C. Cells corresponding to 0.1 OD_600_ were used for growth analysis in liquid medium containing MND or AMB in the presence or absence of doxycycline (50 μ*g*/ml) using a 96-well plate. The OD_600_ was taken for a period of 24 to 48 h using the SpectraMax ID5 microplate reader.

### Drug susceptibility assays.

Susceptibility to antifungal drugs was estimated by either broth microdilution or serial dilution spot assay. The broth microdilution method was used to determine MICs of different antifungals to C. albicans strains. Twofold serial dilutions of antifungal drugs were performed in medium, and 10^4^ cells were inoculated into it. Cells were allowed to grow for 48 h at 30°C, and then absorbance was taken at a 600-nm wavelength (OD_600_). OD_600_ in the presence of drug was then normalized with OD_600_ of YPD medium without antifungal treatment. The relative growth was plotted as percent change in growth. The MIC was then defined at the lowest concentration inhibiting growth by at least 80% relative to the drug-free YPD control after incubation. Serial dilution spot assay was performed by 10-fold serially diluting cells in 0.9% saline, and then a 3-μl aliquot was spotted on YPD agar plates with and without drug. Plates were grown at 30°C, and differences in the growth were photographed after 48 h. Each experiment was repeated three times.

### *In vitro* stress assays.

To study the effect of *in vitro* stresses on AS, C. albicans cells were exposed to indicated stresses like drug stress and iron deficiency. C. albicans (TW1) was grown in YPD medium overnight at 30°C. Cells corresponding to 0.1 OD_600_ were inoculated into fresh YPD medium. Cells were allowed to grow until OD_600_ reached 0.6 to 0.8. For drug stress, cells were exposed to subinhibitory concentrations of drugs (AMB and CAS) and BPS, and samples were collected at different intervals of time (0, 30, 60, and 90 min). RNA was extracted using the Qiagen RNeasy minikit per the manufacturer’s guidelines. Five micrograms of RNA was treated with DNase, and 2 μg DNase-treated RNA was used to synthesize cDNA. cDNA was diluted and used for semiquantitative RT-PCR with specific primers listed in Table S2.

### ROS measurement.

Intracellular reactive oxygen species (ROS) was measured using fluorescent dye, dichlorodihydrofluorescein diacetate (H_2_DCFDA) as previously described (65). Briefly, log-phase cells corresponding to 0.6 OD_600_ were treated with either AMB or MND for 1 h. Cells were then collected and washed with phosphate-buffered saline (PBS) three times. Then, cells corresponding to 2 OD_600_ were incubated with 20 μg H_2_DCFDA for 30 min. One-hundred-microliter cell suspensions were then taken into a 96-well plate, and fluorescence was measured with excitation at 485 nm and emission at 520 nm using a SpectraMax ID5. Each experiment was performed in triplicates. Basal values were subtracted from DCFDA-treated values and then plotted.

### Western blotting.

Overnight cultures were diluted in YPD to 0.1 OD_600_ and then grown until reaching 0.6 OD_600_. MND or AMB was added to the culture and grown for 24 h. Proteins were extracted using the TCA method (66). Extracts corresponding to OD_600_ 1.0 were then separated using 10% SDS-PAGE and blotted onto a nitrocellulose membrane. Rabbit monoclonal anti-GFP antibodies from Cell Signaling Technology (CST) were used to analyze the expression of Sod3-GFP fusion proteins. The membranes were imaged using Odyssey CLx. Anti-PSTAIR antibody was used for a loading control.

### Data availability.

The RNA-Seq files have been submitted to the NBCI SRA database under BioProject no. PRJNA630134. The raw analysis files are given in the supplemental material (Data Sets S1, S2, and S3).
