# Supplementary figures and images for: Identification of Genomewide Alternative Splicing Events in Sequential, Isogenic Clinical Isolates of Candida albicans Reveals a Novel Mechanism of Drug Resistance and Tolerance to Cellular Stresses
Source: mSphere. 2020 Aug 12;5(4):e00608-20. doi: 10.1128/mSphere.00608-20 (PMC7426172; doi:10.1128/mSphere.00608-20)

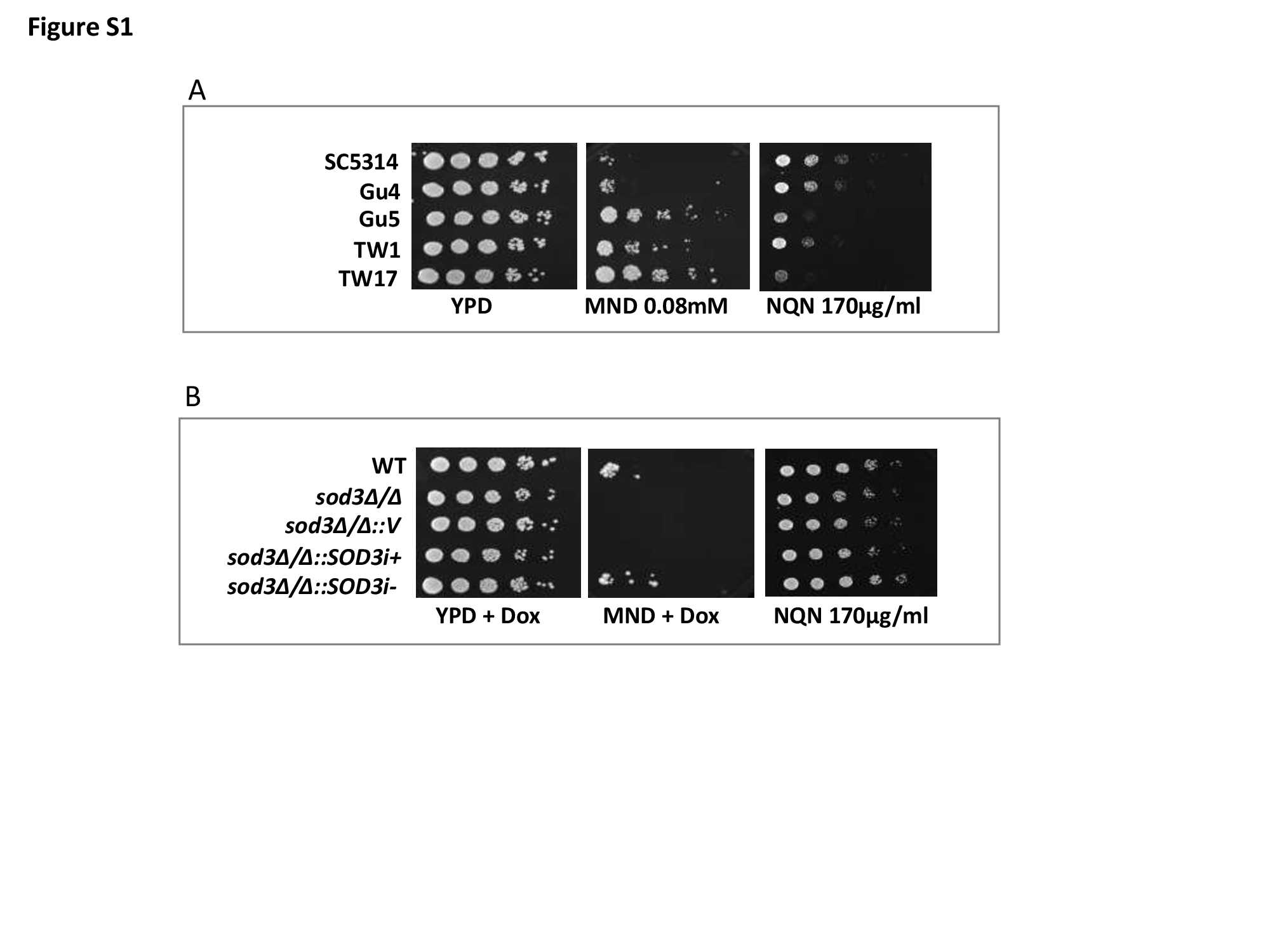

Supplement: FIG S1 [file mSphere.00608-20-sf001.tif]

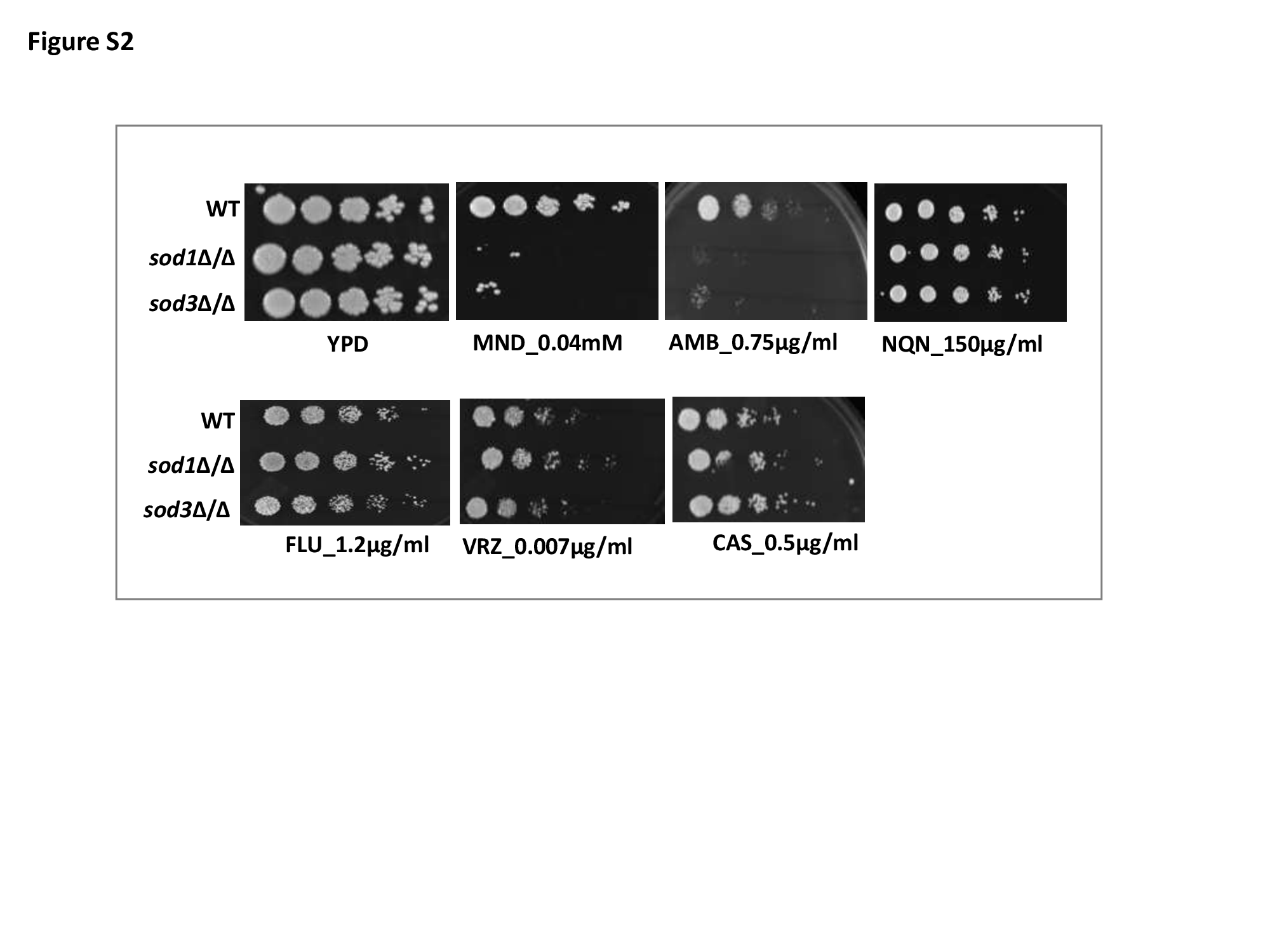

Supplement: FIG S2 [file mSphere.00608-20-sf002.tif]

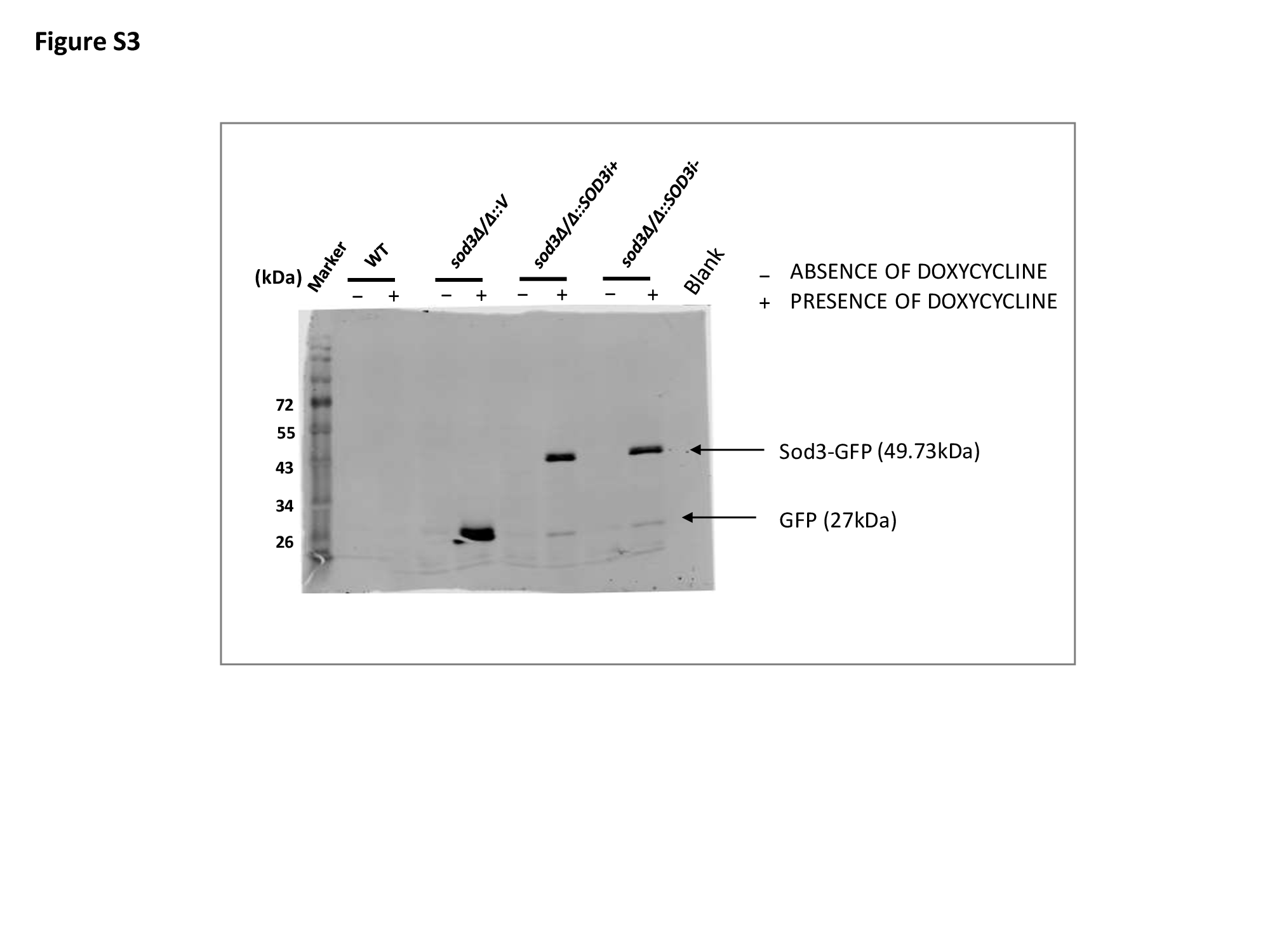

Supplement: FIG S3 [file mSphere.00608-20-sf003.tif]

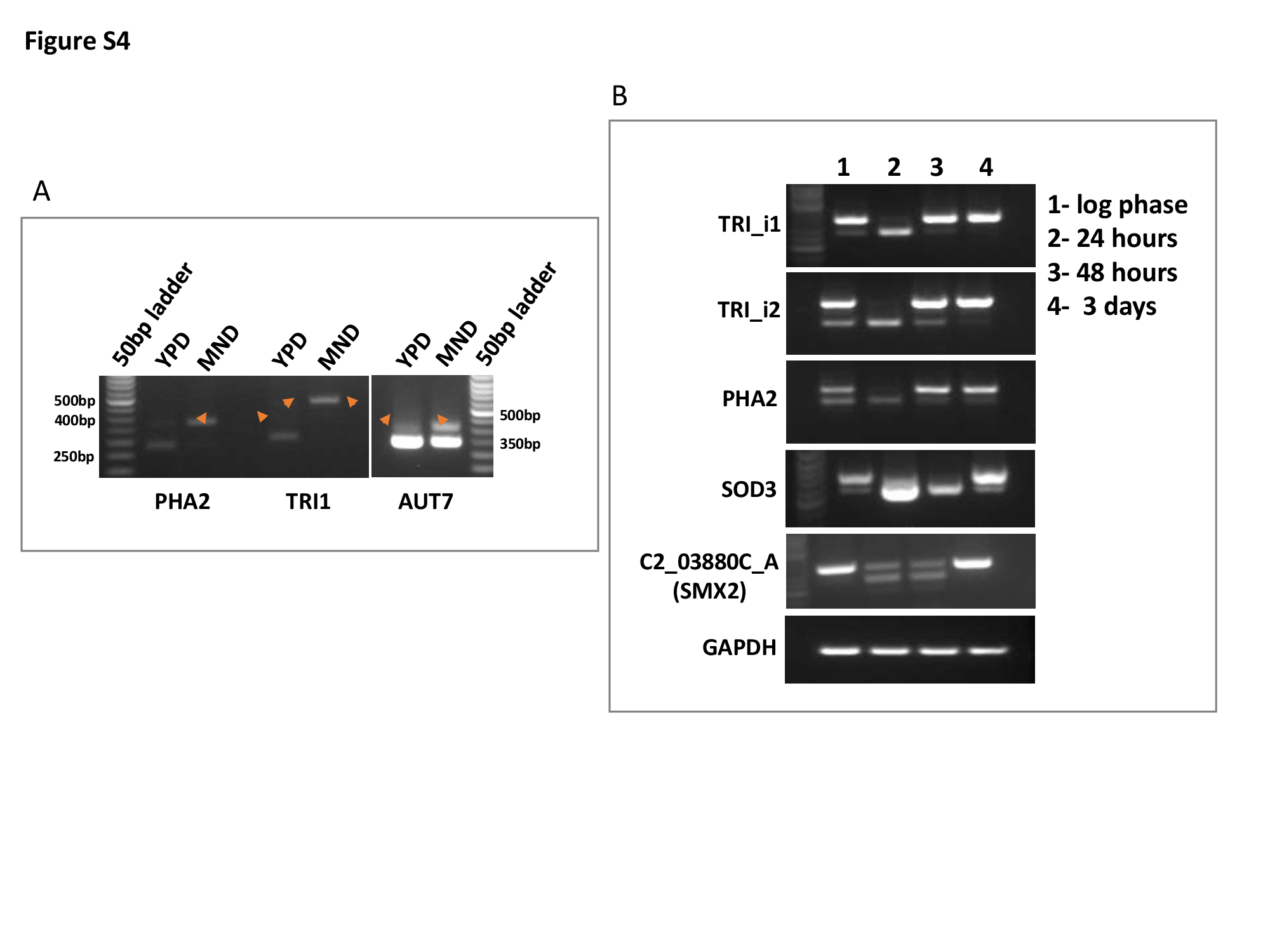

Supplement: FIG S4 [file mSphere.00608-20-sf004.tif]
