# Supplementary material for: Identification of Genomewide Alternative Splicing Events in Sequential, Isogenic Clinical Isolates of Candida albicans Reveals a Novel Mechanism of Drug Resistance and Tolerance to Cellular Stresses
Source: mSphere. 2020 Aug 12;5(4):e00608-20. doi: 10.1128/mSphere.00608-20 (PMC7426172; doi:10.1128/mSphere.00608-20)
Supplement: TABLE S1 [file mSphere.00608-20-st001.docx]

**Table S1.**

| **Strain** | **Description** | **Reference** |
| --- | --- | --- |
| SC5314 | Wild type | (60) |
| TW1 | Fluconazole-susceptible Clinical (oral) isolate | (32) |
| TW17 | Fluconazole-resistant Clinical (oral) isolate with higher expression of MDR1, CDR1, CDR2, ERG11 | (32) |
| Gu4 | Fluconazole-susceptible clinical isolate | (33) |
| Gu5 | Fluconazole-resistant clinical isolate | (33) |
| SN152 | his1Δ/his1Δ, leu2Δ/leu2Δ, arg4Δ/arg4Δ, URA3/ura3Δ::imm434, IRO1/iro1Δ::imm434 | (61) |
| *sod3*𝛥/𝛥 | SN152, SOD3Δ::cmLEU2/SOD3Δ::cdHIS1 | (35) |
| *sod3*𝛥/𝛥::*SOD3_i+_* | *SOD3*𝛥/𝛥::*P_tet__SOD3_i+_* ; containing the unspliced isoform of *SOD3* gene (where intron is retained) | This study |
| *sod3*𝛥/𝛥::*SOD3_i_ -* | *SOD3*𝛥/𝛥::*P_tet__SOD3_i -_* ; containing the spliced isoform of *SOD3* gene (where intron is spliced out) | This study |
| ***Plasmids*** |  |  |
| *pNIM1* |  | (64) |
