# Supplementary material for: Identification of Genomewide Alternative Splicing Events in Sequential, Isogenic Clinical Isolates of Candida albicans Reveals a Novel Mechanism of Drug Resistance and Tolerance to Cellular Stresses
Source: mSphere. 2020 Aug 12;5(4):e00608-20. doi: 10.1128/mSphere.00608-20 (PMC7426172; doi:10.1128/mSphere.00608-20)
Supplement: TABLE S2 [file mSphere.00608-20-st002.docx]

**Table S2.**

| **Genes** | **Primers** |
| --- | --- |
| CR_06850C_A | FP: 5’ TTTCTCCTACTAACGCTCTCAAG 3’ |
|  | RP: 5’ GCCATAATAAAACAACCTAA 3’ |
| *TRI1* | FP: 5’ TTATCTCCAGAATTAACCAA 3’ |
|  | RP: 5’ CAGGATCTTCTTCAGTCATA 3’ |
| *PHA2* | FP: 5’ AATTTTGGAAGCATTAGTTA 3’ |
|  | RP: 5’ TTTCTAGGACCAGAGGGAACA 3’ |
| *ARC19* | FP: 5’ ATGGTACGTAATTTCACTAGC 3’ |
|  | RP: 5’ TTCTGCTCTACTAGTTAAAAATCT 3’ |
| *INO4* | FP: 5’ AGTCGTCCGTTTTATCAG 3’ |
|  | RP: 5’ TCCCGAATATATTAACTCAG 3’ |
| *MTLA1* | FP1: 5’ACGAGTCTTTGGATAGATTA 3’ |
|  | FP2: 5’ TATTCGAGAAGAACAAACAG 3’ |
|  | RP: 5’AACTTGATTTTGCTTTCAG 3’ |
| CR_03310C_A | FP: 5’ GAGCACTTGTACCCATTAG 3’ |
|  | RP: 5’ CCAATAAAAATAGAGGACAA 3’ |
| C1_10750C_A | FP: 5’ AATCGAATTTGAGAGTTATC 3’ |
|  | RP: 5’ GTAACTGAGCTAAATACGTGA 3’ |
| *SOD3* | FP: 5’ ATTGATTGGGCTCTTGATGC 3’ |
|  | RP: 5’ CACGTTCCAAATTGCTTTGA 3’ |
| *AUT7* | FP: 5’ CAATTCAAAGACGAGCATCC 3’ |
|  | RP: 5’ TCGCCAAAAGTATTCTCTCCA 3’ |
| *PRE6* | FP: 5’ GTGCATTATCAATTTTCTCGTATG 3’ |
|  | RP: 5’TTGTGGGATTGGGCTTCTAC 3’ |
| *ACT1* | FP: 5’ AAGCCCAATCCAAAAGAGGT 3’ |
|  | RP: 5’ TGGAAACGTAGAAAGCTGGAA 3’ |
| *PAT1* | FP: 5’ CAGCAACTGATTTATCGGAATGG 3’ |
|  | RP: 5’ ACATCTTCAGGGTTAGGTGG 3’ |
| *TRI1* (i2) | FP: 5’ TGTCTACCATTAAGTTTGATT 3’ |
|  | RP: 5’ CCTACCCATTTCTTCTCTCA. 3’ |
| [C2_03880C_A](http://www.candidagenome.org/cgi-bin/locus.pl?locus=C2_03880C_A)  (*SMX2*) | FP: 5’ CGGAACCAGAATTGAAAAGTG 3’ |
|  | RP: 5’ CAATTGAGTTACCCCTAATGACC. 3’ |
