# Supplementary material for: Identification of Genomewide Alternative Splicing Events in Sequential, Isogenic Clinical Isolates of Candida albicans Reveals a Novel Mechanism of Drug Resistance and Tolerance to Cellular Stresses
Source: mSphere. 2020 Aug 12;5(4):e00608-20. doi: 10.1128/mSphere.00608-20 (PMC7426172; doi:10.1128/mSphere.00608-20)
Supplement: TABLE S3 [file mSphere.00608-20-st003.docx]

**Table S3.**

| **Gene** | **Primers** |
| --- | --- |
| SOD3A | FP:  5’ TTCTCCCTTACTCATCTTGTCGACCGCAGAGTATCTTGACTCTGCCT 3’ |
|  | RP:  5’ CACATACAAATATAAATAGTCGACATGATTACCGAAAACGAAAAGATATCC 3’ |
